# Low Expression of GLIS2 Gene Might Associate with Radiosensitivity of Gastric Cancer

**DOI:** 10.1155/2019/2934925

**Published:** 2019-06-09

**Authors:** Haitong Sun, Jincheng Gu, Zhongyang Li, Qianqian Liu, Jiaxi Lin, Ye Tian, Jianping Cao, Hualong Qin, Zaixiang Tang

**Affiliations:** ^1^Department of Biostatistics, School of Public Health, Medical College of Soochow University, Suzhou 215123, China; ^2^Jiangsu Key Laboratory of Preventive and Translational Medicine for Geriatric Diseases, Medical College of Soochow University, Suzhou 215123, China; ^3^Department of Radiotherapy & Oncology, The Second Affiliated Hospital of Soochow University, Suzhou 215123, China; ^4^School of Radiation Medicine and Protection and Collaborative Innovation Center of Radiation Medicine of Jiangsu Higher Education Institutions, Soochow University, Suzhou 215006, China; ^5^State Key Laboratory of Radiation Medicine and Protection, Soochow University, Suzhou 215123, China; ^6^Department of Thoracic Surgery, The First Affiliated Hospital of Soochow University, Suzhou 215123, China

## Abstract

Human gene GLIS family zinc finger 2 (GLIS2) is a member of GLI-similar zinc finger protein family. Previous studies indicated GLIS2 gene involved in tumorigenesis mechanisms. However, the association between GLIS2 expression and radiosensitivity of gastric cancer has not been well understood. In this study, we used the gastric cancer database in TCGA, and significant association was observed between the low expression of GLIS2 and radiosensitivity of patients with gastric cancer. The adjusted HR values for radiotherapy were 0.162(0.035-0.756) and 0.089(0.014-0.564), with p values 0.021 and 0.010, respectively, in training and testing data, for these patients with low expression of GLIS2, while for patients with high expression of GLIS2, there was no significant survival difference between radiotherapy and nonradiotherapy groups. The adjusted HR were 0.676(0.288-1.586) and 0.508(0.178-1.450), with p values 0.368 and 0.206 in training and testing data, respectively. Further study showed that, for low expression patients, radiotherapy did not significantly increase new tumor event rate and disease progression rate, which partially supported our assumption. These results suggested that low expression of GLIS2 might significantly associate with the radiosensitivity of patients with gastric cancer. The GLIS2 gene might be a potential effective molecular marker of gastric cancer for precise radiotherapy.

## 1. Introduction

Gastric cancer accounts for a large proportion of cancer death worldwide. GLOBOCAN2018 data showed that over 1000,000 people were newly diagnosed with gastric cancer in 2018 [1], nearly 783,000 people died. According to Chinese cancer statistics in 2015 [2], gastric cancer is one of the top four common malignant tumors in China, with the second highest morbidity and mortality. Clinically, the treatment of gastric cancer mainly focuses on surgery, radiotherapy, chemotherapy, immunotherapy, and combined treatment of traditional Chinese medicine as adjuvant treatment methods. Based on extensive application of radiotherapy in clinical practice worldwide, many researchers have paid great attention to how to utilize radiotherapy to improve the life quality of patients with gastric cancer in different directions. Chung et al. did retrospective analysis on the efficacy of treatment of gastric cancer by volumetric-modulated arc therapy (VMAT) and found that VMAT could effectively reduce the treatment time and the irradiation dose at the liver and kidney to alleviate the toxicity of radiotherapy [3]. Some clinical trials had focused on providing optimal radiotherapy strategies for patients with different stages of gastric cancer [4–7]. However, they did not reach consistent results.

Due to diverse sensitivity to radiotherapy in individuals, clinical doctors would like to screen radiosensitive patients who can obtain higher survival benefits. Based on the genome sequence technology, researchers could find potential biomarkers to predict radiosensitive patients, then oncologists and surgeons could adjust their treatment strategy to reduce adverse reactions and improve radiotherapy efficacy [8]. Therefore, it is particularly imperative to find gene biomarkers that can accurately and sensitively predict whether patients with gastric cancer are sensitive to radiotherapy, so as to provide evidence to formulate targeted radiotherapy regimens.

GLIS2 gene is a dual-function transcriptional regulator; its regulation would play an important role during embryonic development [9]. GLIS2 gene could regulate self-renewal capacity in hematopoietic progenitor cells and promote differentiation of megakaryocytes [10, 11], which was identified as one of several genes required for optimal repopulation [12]. Nevertheless, overexpression of human GLIS2 had a negative effect on reprogramming [13, 14], leading to a decreased number of ESC-like colonies, denoting that GLIS2 gene might be associated with cancers. Moreover, expression levels of GLIS2 gene make crucial contribution to maintaining normal renal structure and function [15]; previous studies reported that loss of GLIS2 could lead to increased renal cell apoptosis and fibrosis in human and mice [16]. Mutant mice lacking GLIS2 function showed anterior bowel defects, including esophageal and tracheal stenosis, as well as pulmonary hypoplasia and pulmonary function defects [17].

Recent study had shown that overexpression of GLIS2 had significant association with chemoresistance of gastric cancer [18]. Relationship between the expression of GLIS2 and the sensitivity of radiotherapy for patients suffering from gastric cancer has not been well studied. We assumed the expression level of GLIS2 associated with radiosensitivity of patients. Sensitive patients could obtain survival benefits after radiotherapy. To verify this hypothesis, we analyzed the relationship between GLIS2 expression and radiotherapy sensitivity based on gastric cancer data from TCGA, to provide references for clinical treatment of gastric cancer.

## 2. Data Sources and Methods

### 2.1. Data Sources

In the present study, we analyzed normalized mRNA sequencing data of GLIS2 of the patients with gastric cancer. The data was downloaded in December 2016 from TCGA (The Cancer Genome Atlas, http://cancergenome.nih.gov/) [19, 20], by using TCGA assembler [21]. To clean data, we combined the clinical survival information from several raw data files and eliminated the data of patients with no survival time or survival outcome to obtain effective patient survival information. Then we screened the clinical factors needed among the available data and combined them to obtain a complete clinical data file. In addition, we kept the patients with clear information on radiotherapy. Furthermore, we deleted repeated expression information of normal tissue and combined the mRNA sequencing data of GLIS2 with the clinical data obtained in previous steps to obtain the data used for the present study, which contained 371 patients.

### 2.2. Analysis Method

**Figure 1:**
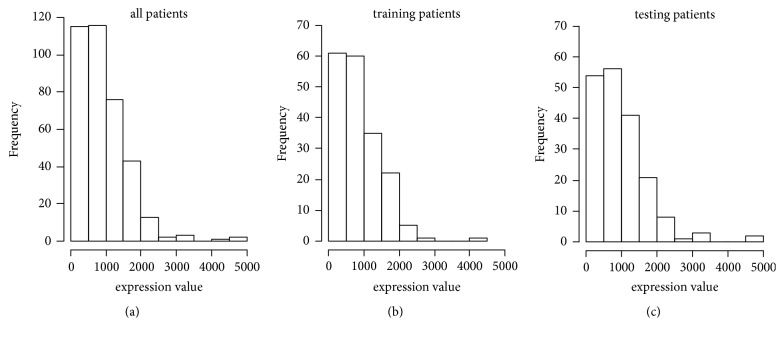
Expression distribution of GLIS2 gene of patients with gastric cancer. (a) Expression distribution of GLIS2 gene in all data. (b) Expression distribution of GLIS2 gene in training data. (c) Expression distribution of GLIS2 gene in testing data.

In the present study, radiotherapy sensitivity was defined as the improved survival benefits of the patients receiving radiotherapy, compared with the patients who did not receive radiotherapy [22, 23]. The gene that can predict individual radiosensitivity could be a potential biomarker for radiosensitivity prediction. In order to validate the hypothesis of this study, we randomly split the data into training data and testing data. Firstly, we generated random number between 0 and 1 for all patients, by using the R function runif(). Then, we picked up a half of patients with small random number, as training data. The rest patients were treated as testing data. The same analysis was performed for both training and testing data. Since the expression level of GLIS2 gene showed skewness distribution in the training data (Figure 1), the median values in the training data were defined as the critical threshold of high and low expression. Then, univariate and multivariate Cox regression analysis were performed for patients with high and low expression. Supplemental Table S1 showed the basic patient characteristics.

In the present study, survival analysis model of R packages survival was adopted for analysis, and survival curves were plotted by R packages rms. P value of 0.05 was taken as the criterion of significance. Missing values were multiple imputed by R package mice [24].

## 3. Results

### 3.1. Correlation Analysis of GLIS2 Expression Level and Clinical Indicators with Survival

In this study, the overall survival was the main observation outcome. The Cox proportional hazard model was used to evaluate the association between GLIS2 expression level and clinical factors with survival. Tables 1 and 2 illustrated the analysis results of training and testing data, respectively. The analysis results showed that radiotherapy could improve the overall survival of patients. However, in both datasets, there were no significant associations between expression level of GLIS2 and overall survival.

These results showed radiation therapy was significantly associated with overall survival. However, we argued that not all patients benefitted from radiation therapy. More accurate radiotherapy can be achieved if sensitive patients are effectively screened. In other words, poorer sensitive patients could be protected from noneffective radiotherapy and the adverse reactions.

### 3.2. Relationship between GLIS2 Expression Levels and Clinical Indicators

We analyzed the relationship between expression levels of GLIS2 and clinical factors by using the chi-square test. The analysis results in Table 3 showed that there were no significant associations between the expressions of GLIS2 and any clinical indicator.

### 3.3. Relationship between Radiotherapy and Survival in High and Low Expression Groups

The main idea of this study was to discuss whether the patients with low expression of GLIS2 were sensitive to radiotherapy. In order to obtain reliable results, we split the data to two part, training data and testing data, and performed survival analysis respectively.  Table 4 demonstrated that, in the training and testing data, for low expression subgroup, there were significant associations between radiotherapy and overall survival. The similar results could be found between univariate and multivariate analysis. The adjusted HR for radiotherapy vs nonradiotherapy were 0.162(0.035-0.756) and 0.089(0.014-0.564) in training and testing data, respectively. For the patients with high expression in training and testing data, radiotherapy could not significantly improve the overall survival, with the adjusted HR 0.676(0.288-1.586) and 0.508(0.178-1.450), respectively.

**Figure 2:**
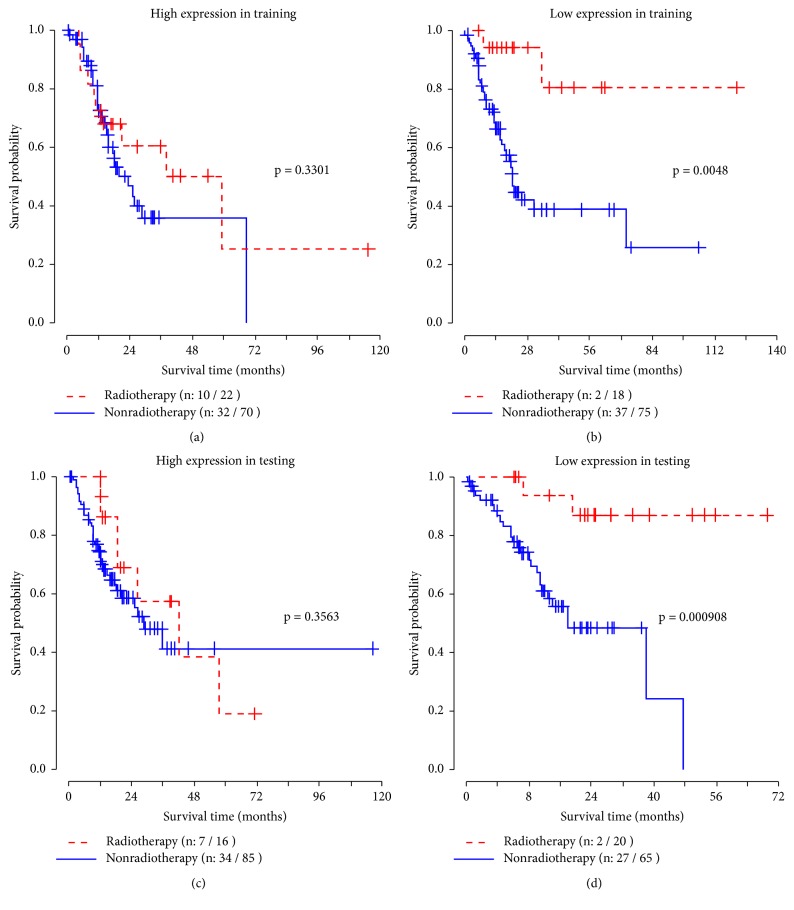
Survival curves under different expression levels of GLIS2 in training and testing data. Log-rank test was used to estimate the P values.

Figure 2 illustrated the survival curves of radiotherapy and nonradiotherapy groups based on different expression levels of GLIS2 gene in the training data and testing data. In the low expression group, the survival time of the patients receiving radiotherapy was significantly prolonged, shown in Figures 2(b) and 2(d). In the high expression group, Figures 2(a) and 2(c), radiotherapy had no significant effect on the overall survival.

We further performed survival analysis by combined training and testing data (Table 4). The same conclusion could be reached. The unadjusted and adjusted HR were 0.694(0.401-1.200) and 0.673(0.360-1.257) for high expression subgroup, respectively. However, for low expression group, the unadjusted and adjusted HR were 0.145(0.053-0.401) and 0.170(0.055-0.521), respectively. For low expression group, radiotherapy exhibited significant clinical efficacy.

**Figure 3:**
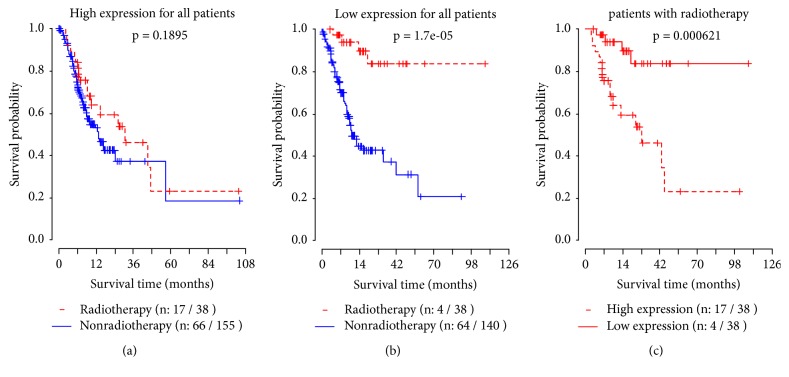
Survival curves under different expression levels of GLIS2 for all patients.

Survival curves for all high and low expression patients were shown in Figures 3(a) and 3(b), respectively.  Figure 3(c) contained the survival curves for patients who received radiotherapy. It can be seen that the survival rate of patients with low expression was significantly prolonged after receiving radiotherapy.

In summary, in the high expression group, patients who received radiotherapy achieved no significant survival benefits than those who were not treated by radiotherapy, while in the low expression group, the survival rate of patients was significantly improved if they received radiotherapy. The results suggested that the low expression of GLIS2 gene might effectively indicate the radiosensitivity of patients. These patients would obtain significant survival benefits from radiotherapy.

### 3.4. Associations among GLIS2 Expressions and Clinical Assessment Factors after Adjuvant Therapy

**Figure 4:**
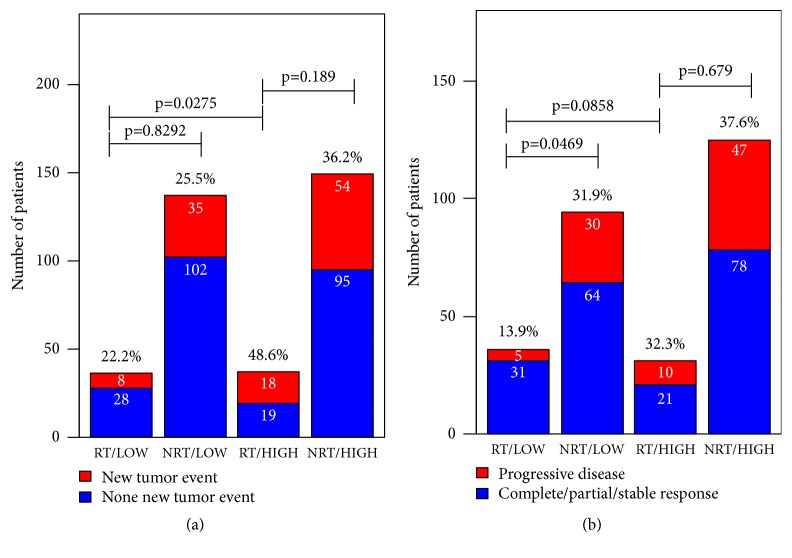
Associations among GLIS2 expressions and clinical assessment factors. Chi-square test was used for comparisons of rates of different groups. RT: radiotherapy; NRT: nonradiotherapy; HIGH: high expression of GLIS2 gene; LOW: low expression of GLIS2 gene.

Figure 4 showed the associations among the expression levels of GLIS2 and two clinical assessment indexes: the new tumor event and progressive disease. There was a significant difference in new tumor event rate between high and low expression group patients under radiotherapy. The low expression group hold lower new tumor event rate 22.2%, which was a half of high expression group (48.6%). Disease progression rate was also approximately significant lower (13.9%) in low expression group, compared with high expression group (32.3%), for patients who received radiotherapy. The results in Figure 4 suggested that, for low expression patients, radiotherapy did not increase new tumor event rate and disease progression rate and even decreased the two rates. These results partially supported that patients with low expression of GLIS2 might be sensitive to radiotherapy.

## 4. Discussion

Radiotherapy is an essential part of adjuvant treatment to cancers, whereas it is also a double-edged sword. It not only kills tumor cells, but also promotes radioimmunity, induces distant metastasis, and damages normal tissues [25]. Studies had reported that increased irradiation had toxic effects on the skin and other normal tissues [26]. Therefore, improving the efficacy of radiotherapy and reducing toxicity had attracted worldwide attention. According to variations of radiosensitivity in individuals, dividing patients with gastric cancer and giving radiation treatment to patients with significant radiotherapy sensitivity would make radiotherapy more accurate, while eliminating the adverse reactions of patients who are not sensitive to radiotherapy after radiotherapy [27]. It can be seen that finding appropriate biomarkers to distinguish sensitive populations is of great importance for clinical treatment. However, effective molecular markers had not been found in gastric cancer so far.

We made use of public data from TCGA in the present study. Due to the difficulties of collecting clinical samples and the large number of potential genes available for external validation, the development of a reliable diagnostic classifier using early nonrandomized phase II data is often not feasible. To overcome these difficulties, we performed an internal validation procedure that randomly divided the data into two parts and analyzed them separately. We found significant associations between expression levels of GLIS2 gene and survival outcome. Low expression of GLIS2 gene might indicate radiosensitivity of patients.  Table S2 illustrated the research results of data. Radiotherapy did have significant effect on improving total survival time of patients suffering from gastric cancer. However, we argued that, in clinical practice, not all patients, some patients with gastric cancer will benefit from radiotherapy. The need for radiation depends on what type of surgery, whether the cancer has spread to somewhere else of body, and in some cases, the age or other clinical factors. If clinical doctors can predict radiosensitivity of patients, they could evaluate sensitive patients more effectively and perform accurate radiotherapy.

In our analysis, we chose median as cutoff to divide high and low expression group. We also performed analysis on other cutoffs, as shown in Figure S1. The results suggested that, when the cutoff larger than median was selected to divide high and low expression group, for patients with high expression, there was no significant survival difference between radiotherapy and nonradiotherapy, while for patients with low expression, patients who received radiotherapy had better survival than who did not receive radiotherapy. We also found that if we selected other cutoffs lower than median, like 10% to 40% quantiles, for these patients with high expression, the HR of radiotherapy were statistically significant, which may be caused by including too many relative lower expression patients. These results were consistent with our conclusion, that low expression patients could be predicted as radiosensitive patients.

It is known that radiotherapy type in gastric cancer includes preoperative, postoperative, and palliative therapy. Preoperative radiotherapy is mainly used in patients with locally advanced gastric cancer to reduce tumor burden and control tumor progression for surgery. TCGA did not provide clear information about radiotherapy type. But, if preoperative therapy was used, the expression data of cancer tissue would be not useful.

Postoperative radiotherapy was the main direction of our research, which is usually combined with chemotherapy to treat patients with resectable gastric cancer as adjuvant therapy. Macdonald et al. conducted Gastrointestinal Cancer Intergroup phase III Trial (INT 0116) and found that postoperative chemoradiotherapy (CRT) could significantly improve the survival rate of patients after radical gastrectomy, though lack of strict trial control [28]. Furthermore, the result of phase III trial led by Lee et al. suggested that additional postoperative radiotherapy to surgery and postoperative chemotherapy could effectively reduce the local recurrence rate and improve the progression-free survival time of patients with positive pathologic lymph nodes [29, 30].

Palliative radiotherapy is mainly applied to the treatment of patients with advanced gastric cancer, focusing on reducing bleeding, pain and other symptoms to improve the quality of life of patients. For M1 stage patients, palliative radiotherapy might be used to improve survival of these patients. We further performed analysis on M0 stage patients and removed M1 stage patients. The results (Table S3) were consistent with our previous results in Table 4. We treated the M stage of patients as a covariate in our analysis.

The mechanisms of the association between GLIS2 and radiotherapy are still not clear. According to the report published by Masetti et al. [31], CBFA2T3-GLIS2 is the most frequent chimeric oncogene identified in non-Down's syndrome acute megakaryocytic leukemia (non-DS-AMKL). It regulated molecules involved in the Hedgehog pathway and Wingless/Integrated (WNT)/*β*-catenin pathways, such as GATA3, HHIP and *β*-catenin. GATA3 was demonstrated to interact with HIF-1*α* to enhance cancer cell invasiveness [32], and inhibition of HHIP promoter methylation suppressed human gastric cancer cell proliferation and migration [33], which would affect the treatment and prognosis of patients with gastric cancer. *β*-catenin regulated cell adhesion to impair DNA repair [34], leading to increased DNA damage and sensitivity of treatment for cancers [35, 36]. GLIS2 could also regulate the interaction between *β*-catenin and T-cell factor/Lymphoid enhancer factor (TCF/LEF) to affect the activation of cyclin D1, which may have association with poor tumor differentiation and prognosis in gastric cancer [37]. These findings suggested that GLIS2 might be an important gene associated with tumor DNA repair and tumor cell cycle. The changes of tumor DNA repair and tumor cell cycle also associated with another important clinical treatment, the radiotherapy. Therefore, GLIS2 may also involve in molecular response under adjuvant radiotherapy. Mechanisms of GLIS2 and radiosensitivity of gastric cancer require further study.

In the present study, expression levels of GLIS2 gene did not associate with overall survival. However, in the subgroup analysis, we came to conclude that gastric cancer patients with low expression of GLIS2 were supposed to possess high radiosensitivity, while patients with high expression of GLIS2 gene were not sensitive to radiotherapy. Population selectivity of radiotherapy has certain guiding value for treatment of gastric cancer.

In clinical work, it was found that the degree of tumor retraction was significantly different after radiotherapy, mainly because of the large individual differences in radiosensitivity. DNA is the main target of ionizing radiation. Cancer risk is usually associated with changes in DNA repair, cell cycle, or apoptotic pathways [38], which plays important roles in radiosensitivity. Gene mutations, polymorphisms, and epigenetic modifications related to DNA repair function can make radiosensitivity variant [39]. In radiotherapy, the survival time varies greatly due to sensitivity of radiotherapy. If cancer patients can be predicted to exhibit radiotherapy sensitivity or resistance, oncologists and surgeons can then alter the treatment to reduce adverse reactions or improve the efficacy of radiotherapy. CBFA2T3-GLIS2 is an important prognostic factor for patients with non-DS-AMKL [10, 40, 41]. Studies had linked the function of GLIS2 to autosomal recessive kidney disease and found that GLIS2 was the most common genetic cause of end-stage renal failure [42]. These studies had demonstrated that the GLIS2 gene had important links to cancers. In our research, we concluded that GLIS2 gene might be an effective molecular marker which was independent of tumor clinical indicators and an indicator of prognostic assessment indexes, which was consistent with the conclusion of previous study [18].

Studies on the association between radiosensitivity of gastric cancer and GLIS2 expression had not been reported before. In this study, internal validation strategy was used to make up for the small sample size and the defects of data only from TCGA. The relationship between GLIS2 and radiosensitivity of gastric cancer was explored, which provided a new reference for clinical improvement of the therapeutic effect on gastric cancer, and an important clue for basic research on radiosensitivity of gastric cancer. Furthermore, we should mention that there were some limitations of this study. We used the data only from TCGA. Sample size was small. In addition, there was no external validation study on our results, like real data from clinical study. Although the limitations existed, the present study still provided a potential helpful clue for further study.

## Figures and Tables

**Table 1:** Associations of clinical indicators and GLIS2 expression level with total survival in training data.

	Univariate analysis		Multivariate analysis	
	HR	P values	HR	P values
Radiotherapy				
Yes	0.458(0.247-0.849)	0.013	0.453(0.226-0.909)	0.026
No	1.000		1.000	
Gender				
Male	1.422(0.886-2.283)	0.145	1.645(0.994-2.724)	0.053
Female	1.000		1.000	
Age				
>60	1.498(0.894-2.509)	0.125	1.569(0.895-2.751)	0.116
≤60	1.000		1.000	
Histologic type				
NOS	0.956(0.556-1.641)	0.868	0.969(0.550-1.706)	0.912
DT/MT/SRT	0.774(0.405-1.480)	0.438	0.724(0.356-1.473)	0.373
PT/TT	1.000		1.000	
T Stage				
T3/T4	1.891(1.088-3.285)	0.024	1.642(0.870-3.098)	0.126
T1/T2	1.000		1.000	
N Stage				
N1/N2/N3	1.837(1.086-3.105)	0.023	1.433(0.666-3.080)	0.357
N0	1.000		1.000	
M Stage				
M1	3.178(1.616-6.250)	0.001	3.305(1.435-7.614)	0.005
M0	1.000		1.000	
Pathological stage				
III/IV	1.810(1.129-2.902)	0.014	1.268(0.609-2.639)	0.526
I/II	1.000		1.000	
Targeted therapy				
Yes	0.753(0.481-1.176)	0.212	1.010(0.352-2.898)	0.986
No	1.000		1.000	
Chemotherapy				
Yes	0.771(0.497-1.196)	0.246	0.792(0.291-2.160)	0.649
No	1.000		1.000	
GLIS2 expression				
High	1.232(0.794-1.910)	0.352	1.353(0.854-2.144)	0.197
Low	1.000		1.000	

Note: HR: hazard ratio; NOS: not otherwise specified; DT: diffuse type; MT: mucinous type; SRT: signet ring type; PT: papillary type; TT: tubular type.

**Table 2:** Associations of clinical indicators and GLIS2 expression level with total survival in testing data.

	Univariate analysis		Multivariate analysis	
	HR	P values	HR	P values
Radiotherapy				
Yes	0.334(0.161-0.690)	0.003	0.309(0.128-0.744)	0.009
No	1.000		1.000	
Gender				
Male	1.100(0.668-1.813)	0.708	1.193(0.711-2.003)	0.504
Female	1.000		1.000	
Age				
>60	1.309(0.793-2.160)	0.293	1.346(0.794-2.282)	0.270
≤60	1.000		1.000	
Histologic type				
NOS	1.521(0.811-2.853)	0.191	1.843(0.962-3.534)	0.065
DT/MT/SRT	1.065(0.520-2.180)	0.863	1.570(0.739-3.333)	0.240
PT/TT	1.000		1.000	
T Stage				
T3/T4	1.700(0.909-3.183)	0.097	0.994(0.472-2.091)	0.986
T1/T2	1.000		1.000	
N Stage				
N1/N2/N3	2.227(1.169-4.240)	0.015	2.280(0.995-5.228)	0.051
N0	1.000		1.000	
M Stage				
M1	1.167(0.559-2.439)	0.681	1.162(0.536-2.520)	0.704
M0	1.000		1.000	
Pathological stage				
III/IV	1.952(1.162-3.279)	0.012	1.703(0.831-3.490)	0.146
I/II	1.000		1.000	
Targeted therapy				
Yes	0.580(0.356-0.946)	0.029	0.997(0.340-2.924)	0.996
No	1.000		1.000	
Chemotherapy				
Yes	0.608(0.376-0.982)	0.042	0.679(0.249-1.856)	0.451
No	1.000		1.000	
GLIS2 expression				
High	1.401(0.869-2.260)	0.166	1.184(0.725-1.936)	0.500
Low	1.000		1.000	

Note: abbreviations were the same with Table 1.

**Table 3:** Relationship between expression levels of GLIS2 and clinical indicators.

	Training data(n=185)				Testing data(n=186)			
	High	Low	*χ* ^2^	P values	High	Low	*χ* ^2^	P values
Gender			0.264	0.608			0.978	0.323
Male	56	61			71	53		
Female	36	32			30	32		
Age			0.000	1.000			2.287	0.131
>60	64	63			59	59		
≤60	28	28			42	25		
Histologic type			5.681	0.058			7.173	0.028
NOS	44	54			50	42		
MT/DT/SRT	30	16			33	17		
PT/TT	18	22			16	26		
T Stage			1.518	0.218			0.080	0.777
T3/T4	69	59			77	63		
T1/T2	23	31			23	22		
N Stage			0.000	1.000			0.243	0.622
N1/N2/N3	64	63			73	57		
N0	28	29			28	27		
M Stage			1.278	0.258			0.330	0.565
M1	4	9			12	7		
M0	88	84			89	78		
Pathological Stage			0.019	0.891			0.013	0.909
III/IV	48	44			51	42		
I/II	42	42			46	41		

Note: abbreviations were the same with Table 1.

**Table 4:** Association analysis between radiotherapy and survival under different expressions of GLIS2.

Data	GLIS2 expression	Unadjusted(RT vs NRT)		Adjusted(RT vs NRT)	
		HR	P values	HR	P values
Training	High (n=92)	0.694(0.332-1.452)	0.332	0.676(0.288-1.586)	0.368
	Low (n=93)	0.165(0.040-0.686)	0.013	0.162(0.035-0.756)	0.021

Testing	High (n=101)	0.677(0.294-1.558)	0.359	0.508(0.178-1.450)	0.206
	Low (n=85)	0.116(0.027-0.509)	0.004	0.089(0.014-0.564)	0.010

All Data	High (n=193)	0.694(0.401-1.200)	0.191	0.673(0.360-1.257)	0.214
	Low (n=178)	0.145(0.053-0.401)	<0.001	0.170(0.055-0.521)	0.002

Note: adjusted factors: gender, age, histologic type, TNM stage, pathological stage, chemotherapy, and targeted therapy.

## Data Availability

The datasets used in the present study are available from The Cancer Genome Atlas database (http://cancergenome.nih.gov/).

## Supplementary Materials

Supplementary Materials Table S1: basic patient characteristics. Table S2: associations of clinical indicators and GLIS2 expression level with total survival in all data. Table S3: association analysis between radiotherapy and survival under different expressions of GLIS2 for M0 stage patients. Figure S1: the HR values of radiotherapy along with different cutoffs. We chose different quantiles as cutoff to divide high and low expression group for further survival analysis.
