# Supplementary material for: Low Expression of GLIS2 Gene Might Associate with Radiosensitivity of Gastric Cancer
Source: J Oncol. 2019 Jun 9;2019:2934925. doi: 10.1155/2019/2934925 (PMC6590498; doi:10.1155/2019/2934925)
Supplement: Supplementary Materials — Table S1: basic patient characteristics. Table S2: associations of clinical indicators and GLIS2 expression level with total survival in all data. Table S3: association analysis between radiotherapy and survival under different expressions of GLIS2 for M0 stage patients. Figure S1: the HR values of radiotherapy along with different cutoffs. We chose different quantiles as cutoff to divide high and low expression group for further survival analysis. [file 2934925.f1.zip › 2934925.f2.docx]

**Supplementary Materials**

Table S1: Basic patient characteristics

|  | N | Median(range)/ratio |
| --- | --- | --- |
| Age | 368 | 67(30-90) |
| NA | 3 |  |
| Race |  |  |
| Non-White  White | 97  239 | 26.15%  64.42% |
| NA | 35 | 9.43% |
| Gender |  |  |
| Male | 241 | 64.96% |
| Female | 130 | 35.04% |
| Status |  |  |
| Censor | 220 | 59.30% |
| Death | 151 | 40.70% |
| Histologic type |  |  |
| NOS | 190 | 51.21% |
| DT/MT/SRT | 96 | 25.88% |
| PT/TT | 82 | 22.10% |
| NA | 3 | 0.81% |
| T Stage |  |  |
| T3/T4 | 268 | 72.24% |
| T1/T2 | 99 | 26.68% |
| NA | 4 | 1.08% |
| N Stage |  |  |
| N1/N2/N3 | 257 | 69.27% |
| N0 | 112 | 30.19% |
| NA | 2 | 0.54% |
| M Stage |  |  |
| M1 | 32 | 8.62% |
| M0 | 339 | 91.38% |
| Pathological stage |  |  |
| III/IV | 185 | 49.87% |
| I/II | 171 | 46.09% |
| NA | 15 | 4.04% |
| Targeted therapy |  |  |
| Yes | 171 | 46.09% |
| No | 196 | 52.83% |
| NA | 4 | 1.08% |
| Chemotherapy |  |  |
| Yes | 176 | 47.44% |
| No | 195 | 52.56% |
| Radiotherapy |  |  |
| Yes | 76 | 20.48% |
| No | 295 | 79.52% |

Note: Non-White: including Asian, Black or African American and Native hawaiian or other pacific islander; NOS: not otherwise specified; DT: diffuse type; MT: mucinous type; SRT: signet ring type; PT: papillary type; TT: tubular type.

Table S2: Associations of clinical indicators and GLIS2 expression level with total survival in all data

|  | Univariate analysis | | Multivariate analysis | |
| --- | --- | --- | --- | --- |
|  | HR | P values | HR | P values |
| Radiotherapy |  |  |  |  |
| Yes | 0.405(0.254-0.646) | <0.001 | 0.424(0.249-0.722) | 0.002 |
| No | 1.000 |  | 1.000 |  |
| Gender |  |  |  |  |
| Male | 1.243(0.882-1.752) | 0.214 | 1.281(0.905-1.813) | 0.163 |
| Female | 1.000 |  | 1.000 |  |
| Age |  |  |  |  |
| >60 | 1.439(1.007-2.058) | 0.046 | 1.542(1.059-2.246) | 0.024 |
| ≤60 | 1.000 |  | 1.000 |  |
| Histologic type |  |  |  |  |
| NOS | 1.172(0.779-1.765) | 0.446 | 1.259(0.828-1.916) | 0.281 |
| DT/MT/SRT | 0.878(0.544-1.417) | 0.593 | 1.109(0.677-1.817) | 0.680 |
| PT/TT | 1.000 |  | 1.000 |  |
| T Stage |  |  |  |  |
| T3/T4 | 1.814(1.197-2.748) | 0.005 | 1.400(0.879-2.231) | 0.157 |
| T1/T2 | 1.000 |  | 1.000 |  |
| N Stage |  |  |  |  |
| N1/N2/N3 | 1.971(1.314-2.955) | 0.001 | 1.704(0.991-2.931) | 0.054 |
| N0 | 1.000 |  | 1.000 |  |
| M Stage |  |  |  |  |
| M1 | 1.750(1.069-2.865) | 0.026 | 1.520(0.907-2.545) | 0.112 |
| M0 | 1.000 |  | 1.000 |  |
| Pathological stage |  |  |  |  |
| III/IV | 1.876(1.324-2.659) | <0.001 | 1.525(0.935-2.488) | 0.091 |
| I/II | 1.000 |  | 1.000 |  |
| Targeted therapy |  |  |  |  |
| Yes | 0.672(0.484-0.933) | 0.018 | 0.811(0.391-1.684) | 0.574 |
| No | 1.000 |  | 1.000 |  |
| Chemotherapy |  |  |  |  |
| Yes | 0.695(0.503-0.959) | 0.027 | 0.874(0.437-1.745) | 0.702 |
| No | 1.000 |  | 1.000 |  |
| GLIS2 expression |  |  |  |  |
| High | 1.297(0.940-1.789) | 0.114 | 1.268(0.915-1.758) | 0.154 |
| Low | 1.000 |  | 1.000 |  |

Note: HR: hazard ratio; NOS: not otherwise specified; DT: diffuse type; MT: mucinous type; SRT: signet ring type; PT: papillary type; TT: tubular type.

Table S3: Association analysis between radiotherapy and survival under different expressions of GLIS2 for M0 stage patients

|  | GLIS2 expression | Unadjusted (RT vs NRT) | | Adjusted (RT vs NRT) | |
| --- | --- | --- | --- | --- | --- |
|  |  | HR | P values | HR | P values |
| Model 1 | High (n=177) | 0.661(0.368-1.189) | 0.167 | 0.610(0.337-1.101) | 0.101 |
|  | Low (n=162) | 0.117(0.036-0.374) | <0.001 | 0.128(0.039-0.415) | <0.001 |
| Model 2 | High (n=177) | 0.661(0.368-1.189) | 0.167 | 0.518(0.266-1.001) | 0.053 |
|  | Low (n=162) | 0.117(0.036-0.374) | <0.001 | 0.100(0.028-0.361) | <0.001 |

Model 1: Adjusted factors: gender, age.

Model 2: Adjusted factors: gender, age, T stage, N stage, pathological stage, targeted therapy, chemotherapy.


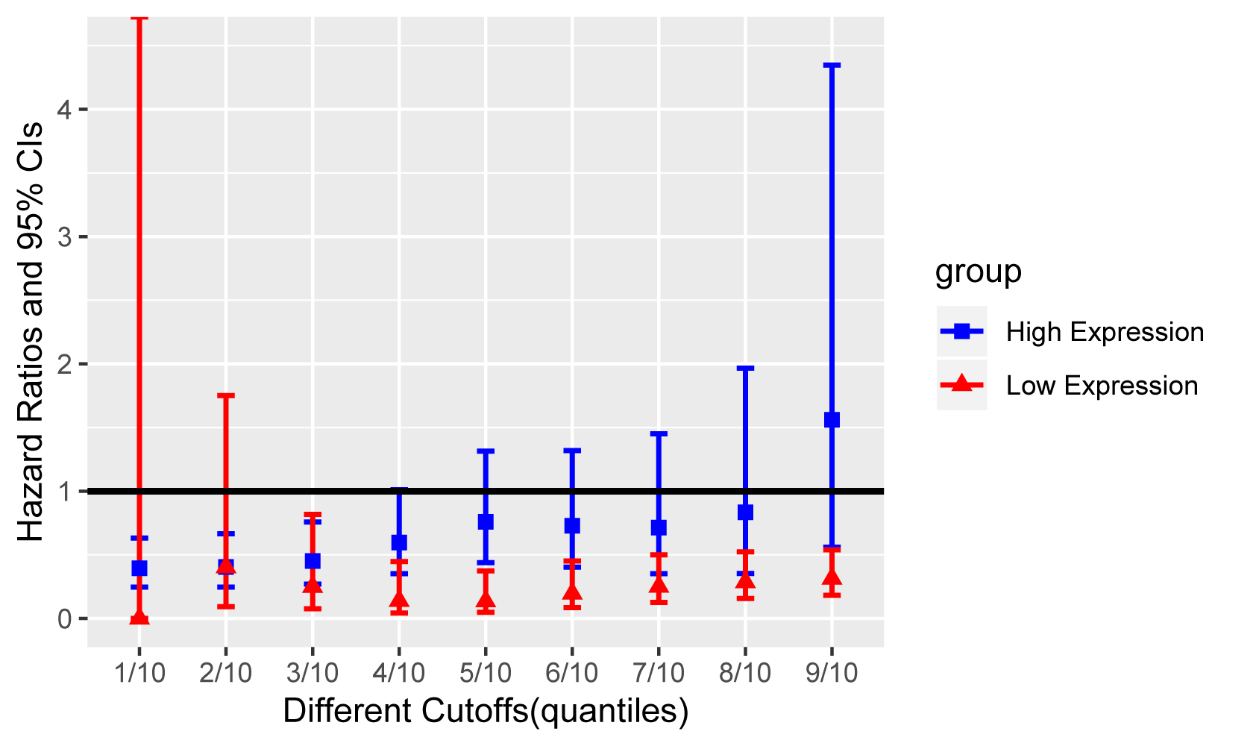


Figure S1: The HR values of radiotherapy along with different cutoffs.
